# A model of disparities: risk factors associated with COVID-19 infection

**DOI:** 10.1186/s12939-020-01242-z

**Published:** 2020-07-29

**Authors:** Yelena Rozenfeld, Jennifer Beam, Haley Maier, Whitney Haggerson, Karen Boudreau, Jamie Carlson, Rhonda Medows

**Affiliations:** 1 Providence Health System (Providence), 1801 Lind Avenue S.W. Valley Office Park - Morin Bldg, 1st Floor Renton, Washington, 98057-9016 USA; 2 Providence Health System (Providence) and Ayin Health Solutions, 1801 Lind Avenue S.W. - Valley Office Park - Gamelin Bldg, Executive Office, Renton, Washington, 98057-9016 USA

**Keywords:** Social determinants of health, Multivariable model, Risk factors, COVID-19, Disparities, Infection

## Abstract

**Background:**

By mid-May 2020, there were over 1.5 million cases of (SARS-CoV-2) or COVID-19 across the U.S. with new confirmed cases continuing to rise following the re-opening of most states. Prior studies have focused mainly on clinical risk factors associated with serious illness and mortality of COVID-19. Less analysis has been conducted on the clinical, sociodemographic, and environmental variables associated with initial infection of COVID-19.

**Methods:**

A multivariable statistical model was used to characterize risk factors in 34,503cases of laboratory-confirmed positive or negative COVID-19 infection in the Providence Health System (U.S.) between February 28 and April 27, 2020. Publicly available data were utilized as approximations for social determinants of health, and patient-level clinical and sociodemographic factors were extracted from the electronic medical record.

**Results:**

Higher risk of COVID-19 infection was associated with older age (OR 1.69; 95% CI 1.41–2.02, *p* < 0.0001), male gender (OR 1.32; 95% CI 1.21–1.44, *p* < 0.0001), Asian race (OR 1.43; 95% CI 1.18–1.72, *p* = 0.0002), Black/African American race (OR 1.51; 95% CI 1.25–1.83, *p* < 0.0001), Latino ethnicity (OR 2.07; 95% CI 1.77–2.41, *p* < 0.0001), non-English language (OR 2.09; 95% CI 1.7–2.57, *p* < 0.0001), residing in a neighborhood with financial insecurity (OR 1.10; 95% CI 1.01–1.25, *p* = 0.04), low air quality (OR 1.01; 95% CI 1.0–1.04, *p* = 0.05), housing insecurity (OR 1.32; 95% CI 1.16–1.5, *p* < 0.0001) or transportation insecurity (OR 1.11; 95% CI 1.02–1.23, *p* = 0.03), and living in senior living communities (OR 1.69; 95% CI 1.23–2.32, *p* = 0.001).

**Conclusion:**

sisk of COVID-19 infection is higher among groups already affected by health disparities across age, race, ethnicity, language, income, and living conditions. Health promotion and disease prevention strategies should prioritize groups most vulnerable to infection and address structural inequities that contribute to risk through social and economic policy.

## Background

As U.S. states begin to reduce coronavirus social restrictions, the risk of contracting COVID-19 is likely to increase. While statistical models have been built to predict severity of illness and mortality related to COVID-19 infection [1], less has been done to predict the risk of initial infection in community settings. Studies to date have contained limited demographic information, have focused on hospitalized patients, and have not been representative of U.S. populations [2–7].

Most studies are limited to known clinical risk factors for severe illness and mortality, such older age [3, 4] and chronic health conditions such as hypertension [3], cardiovascular disease [4], and diabetes [7]. More recent research by the U.S. Centers for Disease Control and Prevention (CDC) has identified specific groups at higher risk for severe illness, such as older adults living in long term care facilities, those with a BMI of forty or higher, and immunosuppressed individuals, including people withHIV/AIDS [8]. However, most risk models have not incorporated clinical, sociodemographic, and environmental variables, which may be predictive of community spread within the U.S.

As with other infectious diseases, predictors of COVID-19 infection may include employment status, education level, income, and housing conditions [9], which could influence the ability to seek care, adhere to treatment, and practice physical distancing measures. Thus, effective strategies for predicting risk factors for community transmission should include both clinical and social factors [10]. The latter factors in particular remain understudied, especially among communities of lower socioeconomic status [10].

Emerging data already show that communities of color and/or low socioeconomic status are experiencing disproportionate rates of serious illness if infected, due to pre-existing economic and health inequities [11, 12].

By performing large scale analyses, healthcare systems can play a role in investigating patient and population differences in disease susceptibility, distinct from mortality risk. The purpose of this study was to use collated data from an entire health system to identify the apparent sociodemographic and environmental, as well as clinical predictors of the risk of COVID-19 infection and their relevance to persistent health disparities across race, ethnicity, socioeconomic status, language, and age [13].

## Methods

### Study design and setting

This study was conducted at Providence Health System, the third largest not-for-profit health system in the U.S., servicing more than five million people across seven states located in the Western and Southwestern portion of the U.S.

### Data source

Data were collected from the Providence enterprise data warehouse. The data elements that were collected were informed by a comprehensive review of prior scientific studies that documented mortality risk factors and the CDC list of groups at higher risk for severe illness [8]. Variables included patient demographic, social, and behavioral history information; chronic conditions documented in clinical history; current conditions; prescribed medications; laboratory testing results; and acute and ambulatory healthcare utilization.

To study sociodemographic and environmental variables, electronic medical record (EMR) data was utilized to link patients’ locations to the U.S. Census Bureau’s 2018 American Community Survey and the CDC air quality data. To join these datasets to EMR data, patient addresses were geocoded, and matched at the census block group or tract level.

Glottolog, a repository for the world’s languages, was used to assign language groups. Geographic regions and clinical symptoms were also included as variables. Census data on educational attainment and financial insecurity were used to assess socioeconomic status.

### Participants and procedures

Patients residing in Alaska, Washington, Oregon, Montana, and California (Los Angeles and parts of Orange County) who were tested for acute respiratory syndrome coronavirus 2 (SARS-CoV-2) infection between February 28, 2020 and April 27, 2020 were included in the data set. Testing mechanisms included swabs from respiratory specimens appropriate for viral RNA testing from eight testing platforms.

### Outcomes and predictors

The principle dependent variable for our model was COVID-19 infection, as indicated by a positive lab test.

Distributions of all continuous variables including age, BMI, number of medications, and neighborhood financial insecurity were examined for normality and transformed into categorical attributes. Comorbidities were determined by problem list documentation or clinical encounter diagnoses using standard International Classification of Diseases, Tenth Revision, Clinical Modification (ICD-10-CM) nomenclature and further summarized into a measure of disease severity using total number of chronic conditions. Substance, tobacco, and alcohol consumptions were captured from social history assessments and clinician documentation.

The following variables were used as indicators of physical proximity to other people (i.e., structural barriers to social distancing): transportation insecurity, relationship status, employment, housing insecurity, and age-stratified communal living.

### Statistical methods and modeling

Descriptive statistics were used to summarize study participants. Continuous variables were described by means and standard deviations, while categorical variables were described using frequencies and percentages. We conducted bivariate analysis to assess a significant effect of each factor on the outcome. All covariates with *p* < 0.25 in the bivariate analysis were considered for model inclusion since use of a more traditional level of 0.05 often fails to identify variables whose association with the outcome could become stronger in the presence of other variables [14]. In addition, all variables of known clinical importance found in previous studies that could make an important contribution were included to improve upon previous models [1]. Beginning with all variables of interest, a stepwise selection with backward elimination was used to create a multivariable logistic regression model for predicting risk of infection.

Initial parameters for the model were identified in the training set and then tested at the subsequent step, with data randomly partitioned into two independent data subsets: 80% for training and building the model and another 20% for testing. Missing data was recoded as unknown and included in the analysis. Detailed covariate definitions and data sources are shown in the supplement.

The model’s ability to discriminate COVID-19 infection in the validation data set was evaluated using the area under the receiver operating characteristic curve and Hosmer-Lemeshow goodness-of-fit statistic. The observed and expected frequencies within each decile of risk was compared [14]. All data manipulation and modeling were completed in SAS EG (SAS Institute, Carry NC).

For all independent predictor subgroups, the risk of COVID-19 infection was quantified with odds ratios (OR) and 95% confidence intervals. These risks were calculated using the entire data set.

## Results

### Study population

A total of 34,503 COVID-19 tested patients were included in the study (Table 1). The average age was 50 years old (SD 20), 59.6% (21,209) were female, 12% (4183) were identified as non-white race, and 66% (22,610) had at least one comorbidity. Within the study population, 7.5% (2578) patients tested positive and 92.5% (31,925) tested negative for COVID-19. Of patients testing positive, 36% (924) were hospitalized and 9% (240) died during the study period.

**Table 1:** Study Participant Demographics and Characteristic

	Tested patients (***N*** = 34,503)		Tested Positive (***N*** = 2578)		Tested Negative (31,925)	
	N	%	N	%	N	%
**Sociodemographic**						
Age						
< 18	1393	4.0	35	1.4	1358	4.3
18–29	4494	13.0	268	10.4	4226	13.2
30–39	5803	16.8	304	11.8	5499	17.2
40–49	5468	15.8	411	15.9	5057	15.8
50–59	5663	16.4	523	20.3	5140	16.1
60–69	5467	15.8	467	18.1	5000	15.7
70–79	3522	10.2	296	11.5	3226	10.1
80+	2693	7.8	274	10.6	2419	7.6
Gender						
Female	21,209	59.6	1352	52.4	19,219	60.2
Male	13,924	40.4	1225	47.5	12,699	39.8
Education						
Education < 12 years	9565	27.7	826	32.0	8739	27.4
Employment						
Student	1148	3.3	51	2.0	1097	3.4
Employed	16,570	48.0	1311	50.9	15,259	47.8
Not Employed	5872	17.0	362	14.0	5510	17.3
Retired	7284	21.1	637	24.7	6647	20.8
Unknown	3629	10.5	217	8.4	3412	10.7
Race						
White	24,799	71.9	1437	55.7	23,362	73.2
American Indian | Alaska Native	465	1.3	13	0.5	452	1.4
Asian	1713	5.0	209	8.1	1504	4.7
Black | African American	1649	4.8	159	6.2	1490	4.7
Native Hawaiian | Pacific Islander	356	1.0	25	1.0	331	1.0
Unknown	5521	16.0	735	28.5	4786	15.0
Ethnicity						
Other Ethnic Groups	30,938	89.7	1940	75.3	28,998	90.8
Hispanic or Latino	3565	10.3	638	24.7	2927	9.2
Religious Affiliation						
Agnostic	10,938	31.7	661	25.6	10,277	32.2
Christian	14,483	42.0	1219	47.3	13,264	41.5
Other Religion	1181	3.4	103	4.0	1078	3.4
Unknown	7901	22.9	595	23.1	7306	22.9
Relationship						
Single	12,940	37.5	790	30.6	12,150	38.1
Divorced or Legally Separated	5248	15.2	383	14.9	4865	15.2
Married or Significant Other	15,173	44.0	1305	50.6	13,868	43.4
Unknown	1142	3.3	100	3.9	1042	3.3
Language						
English	32,277	93.5	2085	80.9	30,192	94.6
Sino-Tibetan	286	0.8	55	2.1	231	0.7
Spanish	1022	3.0	291	11.3	731	2.3
Other Languages	918	2.7	147	5.7	771	2.4
**Clinical**						
Body Mass Index						
Normal	7088	20.5	444	17.2	6644	20.8
Underweight	554	1.6	30	1.2	524	1.6
Moderately Obese	5667	16.4	452	17.5	5215	16.3
Overweight	8009	23.2	670	26.0	7339	23.0
Severely Obese	3080	8.9	243	9.4	2837	8.9
Very Severely Obese	2835	8.2	208	8.1	2627	8.2
Unknown	7270	21.1	531	20.6	6739	21.1
Number of Chronic Conditions						
0	11,893	34.5	1017	39.4	10,876	34.1
1–2	12,185	35.3	924	35.8	11,261	35.3
3–4	6563	19.0	406	15.7	6157	19.3
5+	3862	11.2	231	9.0	3631	11.4
Clinical Diagnosis						
Diagnosis of Diabetes	4942	14.3	456	17.7	4486	14.1
Diagnosis of Kidney Disease	65	0.2	6	0.2	59	0.2
Diagnosis of HIV/AIDS	141	0.4	13	0.5	128	0.4
Diagnosis of Dementia	1039	3.0	135	5.2	904	2.8
Polypharmacy						
0 Prescriptions	8933	25.9	826	32.0	8107	25.4
1–9 Prescriptions	18,066	52.4	1370	53.1	16,696	52.3
10–19 Prescriptions	5307	15.4	298	11.6	5009	15.7
20–29 Prescriptions	1549	4.5	61	2.4	1488	4.7
30+ Prescriptions	648	1.9	23	0.9	625	2.0
Mental Health and Substance Use						
History of Illicit Drug Use	4375	12.7	137	5.3	4238	13.3
History of Tobacco Use	5606	16.2	162	6.3	5444	17.1
Diagnosis of Serious Persistent Mental Illness	4507	13.1	177	6.9	4330	13.6
Diagnosis of Substance Use Disorder	3605	10.4	112	4.3	3493	10.9
Primary Care Affiliation						
Internal Primary Care Provider	14,682	42.55	894	34.7	13,788	43.2
External Primary Care Provider	12,456	36.1	1026	39.8	11,430	35.8
Unknown Primary Care Provider	7365	21.35	658	25.5	6707	21.0
Electronic Communication through the EMR	22,158	64.2	1337	51.9	20,821	65.2
Symptoms						
Fever	20,565	59.6	1995	77.4	18,570	58.2
Cough	24,506	71.0	2062	80.0	22,444	70.3
Breath	21,587	62.6	1857	72.0	19,730	61.8
Chills	694	2.0	88	3.4	606	1.9
Myalgia	955	2.8	145	5.6	810	2.5
**Environmental**						
Region						
Oregon	10,486	30.4	454	17.6	10,032	31.4
Alaska	1837	5.3	86	3.3	1751	5.5
Puget Sound	6273	18.2	704	27.3	5569	17.4
Southern California	3852	11	605	23.5	3247	10.2
Washington | Montana	12,055	34.9	729	28.3	11,326	35.5
Age-Stratified Communal Living						
Non-Communal Living	24,581	71.2	1766	68.5	22,815	71.5
Adult Community	1619	4.7	143	5.5	1476	4.6
Adult and Youth	5294	15.3	400	15.5	4894	15.3
Multigenerational	1970	5.7	177	6.9	1793	5.6
Senior Living	489	1.4	58	2.2	431	1.4
Other	550	1.6	34	1.3	516	1.6
Financial Insecurity	9993	29.0	768	29.8	9225	28.9
Housing Insecurity	6743	19.5	709	27.5	6034	18.9
Transportation Insecurity	10,429	30.2	810	31.4	9619	30.1
Low Air Quality	9664	28.0	754	29.2	8910	27.9

### Risk factors

Table 2 shows the twenty-nine sociodemographic, clinical, and environmental covariates associated with odds of infection.

**Table 2:** Final Multivariable Model Results

	OR	95% CI	*p*-value
**Sociodemographic**			
Age			
18–29	–	–	–
< 18	0.33	[0.22–0.49]	<.0001
30–39	0.88	[0.73–1.05]	0.1574
40–49	1.27	[1.06–1.52]	0.011
50–59	1.69	[1.41–2.02]	<.0001
60–69	1.65	[1.36–2.01]	<.0001
70–79	1.59	[1.24–2.05]	0.0003
80+	1.64	[1.24–2.17]	0.0005
Gender			
Female	–	–	–
Male	1.32	[1.21–1.44]	<.0001
Education			
Education < 12 years	1.02	[1.01–1.14]	0.0435
Employment			
Student	–	–	–
Employed	1.85	[1.39–2.46]	<.0001
Not Employed	1.41	[1.05–1.91]	0.024
Retired	2.06	[1.54–2.76]	<.0001
Unknown	1.37	[1–1.87]	0.0494
Race			
White	–	–	–
American Indian | Alaska Native	0.63	[0.36–1.12]	0.1156
Asian	1.43	[1.18–1.72]	0.0002
Black| African American	1.51	[1.25–1.83]	<.0001
Native Hawaiian | Pacific Islander	1.02	[0.66–1.57]	0.9438
Unknown	1.34	[1.18–1.52]	<.0001
Ethnicity			
Other Ethnic Groups	–	–	–
Hispanic or Latino	2.07	[1.77–2.41]	<.0001
Religious Affiliation			
Agnostic	–	–	–
Christian	1.28	[1.15–1.43]	<.0001
Other Religion	1.01	[0.77–1.24]	0.1453
Unknown	1.10	[0.97–1.25]	0.8752
Relationship			
Single	–	–	–
Divorce or Legally Separated	1.08	[0.93–1.26]	0.3293
Married or Significant Other	1.12	[1.01–1.25]	0.0357
Unknown	0.96	[0.74–1.24]	0.7468
Language			
English	–	–	–
Sino-Tibetan	1.98	[1.38–2.84]	0.0002
Spanish	1.60	[1.31–1.94]	<.0001
Other Languages	2.09	[1.7–2.57]	<.0001
**Clinical**			
Body Mass Index			
Normal	–	–	–
Underweight	0.80	[0.54–1.2]	0.2857
Moderately Obese	1.25	[1.08–1.45]	0.0033
Overweight	1.28	[1.12–1.46]	0.0003
Severely Obese	1.45	[1.21–1.73]	<.0001
Very Severely Obese	1.58	[1.31–1.91]	<.0001
Unknown	0.99	[0.84–1.16]	0.8867
Number of Chronic Conditions			
0	–	–	–
1–2	0.83	[0.74–0.93]	0.001
3–4	0.63	[0.54–0.74]	<.0001
5+	0.55	[0.44–0.69]	<.0001
Clinical Diagnosis			
Diagnosis of Diabetes	1.40	[1.22–1.61]	<.0001
Diagnosis of Kidney Disease	1.03	[1.01–2.3]	0.0385
Diagnosis of HIV/AIDS	1.43	[1.03–2.63]	0.0252
Diagnosis of Dementia	2.01	[1.61–2.51]	<.0001
Polypharmacy			
0 Prescriptions	–	–	–
1–9 Prescriptions	0.76	[0.68–0.86]	<.0001
10–19 Prescriptions	0.60	[0.5–0.71]	<.0001
20–29 Prescriptions	0.43	[0.32–0.59]	<.0001
30+ Prescriptions	0.42	[0.26–0.66]	0.0002
Mental Health and Substance Use			
History of Illicit Drug Use	0.63	[0.53–0.77]	<.0001
History of Tobacco Use	0.46	[0.38–0.54]	<.0001
Diagnosis of Serious Persistent Mental Illness	0.77	[0.65–0.92]	0.003
Diagnosis of Substance Use Disorder	0.70	[0.56–0.87]	0.001
Primary Care Provider Affiliation			
Internal Primary Care Provider	–	–	–
External Primary Care Provider	1.23	[1.1–1.37]	0.0004
Unknown Primary Care Provider	1.27	[1.11–1.46]	0.0005
Electronic Communication through the EMR	0.72	[0.66–0.8]	<.0001
Symptoms			
Symptoms of Fever	2.39	[2.15–2.65]	<.0001
Symptoms of Cough	1.44	[1.28–1.62]	<.0001
Shortness of Breath	1.34	[1.21–1.49]	<.0001
Symptoms of Chills	1.40	[1.09–1.79]	0.0086
Myalgia	1.80	[1.47–2.2]	<.0001
**Environmental**			
Region			
Oregon	–	–	–
Alaska	1.31	[1–1.7]	0.0469
Puget Sound	2.83	[2.44–3.28]	<.0001
Southern California	2.39	[2.06–2.78]	<.0001
Washington Montana	1.49	[1.29–1.73]	<.0001
Age-Stratified Communal Living			
Non-Communal Living	–	–	–
Adult Community	1.30	[1.07–1.58]	0.0082
Adult and Youth	1.07	[0.95–1.21]	0.2835
Multigenerational	1.07	[0.9–1.28]	0.4563
Senior Living	1.69	[1.23–2.32]	0.0011
Other	1.12	[0.77–1.64]	0.5492
Financial Insecurity	1.10	[1.01–1.25]	0.0392
Housing Insecurity	1.32	[1.16–1.5]	<.0001
Transportation Insecurity	1.11	[1.02–1.23]	0.0285
Low Air Quality	1.01	[1–1.04]	0.0502

#### Sociodemographic risk factors

Comparatively, individuals between 50 and 59 years of age (OR 1.69; 95% CI 1.41–2.02, *p* < 0.0001) or male gender (OR 1.32; 95% CI 1.21–1.44, *p* < 0.0001) were more likely to contract COVID-19. Being employed (OR 1.85; 95% CI 1.39–2.46, *p* = 0.02), or retired (OR 2.06; 95% CI 1.54–2.76, *p* < 0.0001) was associated with higher levels of infection. Asian race (OR 1.43; 95% CI 1.18–1.72, *p* = 0.0002), Black/African American race (OR 1.51; 95% CI 1.25–1.83, *p* < 0.0001), and Latino ethnicity (OR 2.07; 95% CI 1.77–2.41, *p* < 0.0001) were more likely than whites to contract COVID-19. Individuals who identified as being married or having a significant other were at higher infection risk (OR 1.12; 95% CI 1.01–1.25, *p* = 0.04), as were those whose primary language was not English (OR 2.09; 95% CI 1.7–2.57, *p* < 0.0001), and those who self-reported their religious affiliation as Christian denomination (OR 1.28; 95% CI 1.15–1.43, *p* < 0.0001).

#### Clinical risk factors

Clinical risk factors including being very severely obese (OR 1.58; 95% CI 1.31–1.91, *p* < 0.0001), or having been diagnosed with diabetes (OR 1.40; 95% CI 1.22–1.61, *p* < 0.0001), chronic kidney disease (OR 1.03; 95% CI 1.01–2.3, *p* = 0.04), dementia (OR 2.01; 95% CI 1.61–2.51, *p* < 0.0001), or HIV/AIDS (OR 1.43; 95% CI 1.03–2.63, *p* = 0.03). Having an external primary care provider (OR 1.23; 95% CI 1.1–1.37, *p* = 0.0004) or an unknown primary care provider (OR 1.27; 95% CI 1.11–1.46, *p* = 0.0005) were associated with higher infection risk compared to having a primary care provider within the Providence Health System. Receiving electronic communication through the EMR was associated with a lower infection risk (OR 0.72; 95% CI 0.66–0.8, *p* < 0.0001).

#### Environmental risk factors

Patients living in areas with low air quality (OR 1.01; 95% CI 1.0–1.04, *p* = 0.05), financial insecurity (OR 1.10; 95% CI 1.01–1.25, *p* = 0.04), transportation insecurity (OR 1.11; 95% CI 1.02–1.23, *p* = 0.03), or housing insecurity (OR 1.32; 95% CI 1.16–1.5, *p* < 0.0001) were at higher risk of infection. Living in senior living facilities was associated with greater infection risk (OR 1.69; 95% CI 1.23–2.32, *p* = 0.001).

### Prediction of infection risk

The model performed consistently across training and testing data sets with a receiver operating characteristic area under the curve of 0.78 and the Hosmer-Lemeshow chi-square of 4.4 (*p* = 0.81). The probabilities of infection was partitioned into “deciles of risk” (i.e. equal groups from smallest to the largest) did not highlight any “underperforming” areas.

## Discussion

### Clinical risk factors

This retrospective study of the risk of COVID-19 infection identified several clinical risk factors also associated with serious illness in prior studies, including older age [3], male gender [15], diabetes [7], chronic kidney disease [16], high BMI [17], and immunosuppression [18]. However, some factors previously found to increase mortality risk, such as hypertension [3], and cardiovascular disease, liver disease, lung disease, or asthma [8], were not significant factors associated with initial COVID-19 infection.

Surprisingly, being prescribed more than ten medications or having a greater number of chronic conditions was associated with less infection risk, suggesting possible risk reduction behavior based on perceived risk. Further research is needed to understand the differences between factors associated with initial infection risk and those associated with serious illness and mortality once the infection occurs.

Healthcare access through a relationship with an internal primary care provider was associated with a lower infection risk; however, this may be a result of higher rates of testing for COVID-19 compared to individuals with no primary care provider. Patients without a primary care provider may have only been tested for COVID-19 after respiratory and other possible COVID-19 symptoms became conspicuous, thus increasing the probability of a positive test.

Receiving secure electronic communication through the EMR was associated with lower risk of infection, suggesting that access to health advice and education may reduce risk.

Serious mental illness and drug and tobacco use were associated with lower risk; however further study is necessary to understand the mechanisms behind such associations.

#### Sociodemographic risk factors

Race and ethnicity appeared to be important predictors of risk. Higher risk of infection among Black, indigenous, and/or people of color may be associated with other sociodemographic and environmental characteristics found to also be significant in this study. African Americans and Latinos are more likely to live in communities with poor air quality [19], work in jobs that cannot telecommute [20], and lack access to healthcare [21] which may increase the risk of infection and contribute to racial disparities in mortality. Additionally, chronic conditions such as obesity, stroke, and diabetes, and premature death also affect African Americans and Latinos disproportionately compared to whites [13]. Communities of color are also more likely to experience lower socioeconomic status [22], and be employed as essential workers [10]. Additionally, for these and other vulnerable groups, lack of personal transportation is both a barrier to healthcare access [23] and social distancing, further exacerbating infection risk. For these reasons, communities of color experience more structural barriers to social distancing measures and are more vulnerable to severe illness.

Having limited English proficiency can be a barrier to accessing health services and understanding health information, especially when written translations and/or trained translators are not available [24]. Over the course of the pandemic, health information has changed rapidly (e.g., mandates for masking), which can create barriers to accessing information and could leave indigenous and immigrant communities uninformed. During the Ebola epidemic in West Africa, language barriers were an obstacle to slowing the spread of the disease [25]. People with LEP are also more likely to have low health literacy compared to English speakers and are at a higher risk of poor health [26]. Culturally and linguistically appropriate interventions are essential, including communication materials of differentformats and reading levels developed through the collaboration of native language speakers and English speakers, as well as the use of community health workers that can engage with underserved groups [27].

### Environmental risk factors

Older age may be considered both a clinical and an environmental risk factor, as it moderates both comorbidities (e.g., dementia) requiring caregiving and housing situations (e.g., living in senior communities). Our results showed that some sociodemographic patient characteristics that influence environmental exposure to social contact were also associated with increased rates of COVID-19 infection, such as being married or having a significant other, being employed, lacking access to a personal vehicle, and living in overcrowded housing, each of which significantly increased infection risk. Religious affiliation was also associated with increased risk, which may be attributed to attendance of large religious services or other behaviors associated with religious identity.

People experiencing housing insecurity may experience challenges with physical distancing, especially when housing is crowded. These individuals may also lack hand washing facilities and/or running water [28]. Both factors could facilitate community spread of infectious diseases.

Regional differences in infection risk were evident, with Southern California and the Western Washington having the highest infection rates (15.7 and 11.3% of tested patients) while Oregon and Alaska (4.3 and 4.7%) had the lowest rates. These regional differences may reflect some combination of population density, proximity to the initial points of COVID-19 entry into the U.S., and state-specific COVID-19 precautions.

### Study limitations

This study was limited to patient data from the Providence Health System, and publicly available data sets. Although the organization serves a diverse patient population across seven Western U. S states, the generalizability of this study to the entire U.S is unclear. With limited testing available and evolving screening guidelines, clinical discernment and personal bias may have impacted which individuals received testing and thus may have influenced the rates of testing in certain populations. Additionally, it is impossible to correlate patient data to measures of individual patient behaviors, such as mask use or adherence to social distancing recommendations. Finally, this study focused on factors associated with initial infection risk, however other factors may further influence outcomes such as disease severity, time in hospital, and mortality.

## Conclusions

Our construction of a multi-faceted prediction model of COVID-19 infection risk in our large, multi-state population has important implications for healthcare systems, public health departments, and city and state governments to further reduce the risk of infection and prevent the spread of COVID-19 in communities that may be disproportionately impacted. Knowledge of the complex mixture of clinical, ethnic, linguistic, and environmental factors that contribute to infection risk should enable more targeted public health approaches to decrease COVID-19 infection.

Linguistically and culturally appropriate prevention education, healthcare access including routine care and COVID-19 testing, and efforts to address substandard housing and hazardous working conditions are essential to reducing risk among vulnerable groups, especially communities of lower socioeconomic status which experience a greater incidence of infectious diseases [29]. Now, and as communities seek to “re-open,” addressing the disparities in infection that contribute to rates of serious illness and mortality are needed to alleviate the disproportionate burden of the pandemic and persisting health disparities.

## Supplementary information

**Additional file 1.** Model Covariate Definitions and Sources.

## Data Availability

The datasets generated and analyzed during the current study are not publicly available as stipulated by the Providence IRB that all patient level data would reside within Providence secured computer network, only accessible to the study investigators, and locked up on Providence property. The publicly available data source was accessed via a proprietary data vendor, which cannot be shared publicly due to their contractual agreement. The underlying publicly available data sources include the 2018 American Community Survey, the Centers for Disease Control and Prevention Air Quality and Glottolog.

## Abbreviations

BMI: Body Mass Index
CDC: Centers for Disease Control and Prevention
EMR: Electronic Medical Record
OR: Odds Ratio
