# Supplementary material for: A model of disparities: risk factors associated with COVID-19 infection
Source: Int J Equity Health. 2020 Jul 29;19:126. doi: 10.1186/s12939-020-01242-z (PMC7387879; doi:10.1186/s12939-020-01242-z)
Supplement: Supplementary file 1 — Additional file 1. Model Covariate Definitions and Sources. [file 12939_2020_1242_MOESM1_ESM.docx]

**Model Covariate Definitions and Sources**

| Variable | Source | Definition |
| --- | --- | --- |
| **Sociodemographic** | | |
| Age | EMR | Age calculated at the time of study based on the date of birth in the EMR |
| Gender | EMR | Self-reported |
| Education | ACS | Neighborhood education level used as proxy for individuals, since EMR data is so sparsely populated. Assigned via geocoded patient address using percent of population age 25+ with no high school diploma, as defined by ACS |
| Employment | EMR | Self-reported |
| Race | EMR | Self-reported |
| Ethnicity | EMR | Self-reported |
| Religion | EMR | Self-reported |
| Relationship | EMR | Self-reported marital status |
| Language | EMR | Self-reported |
| **Clinical** | | |
| Body Mass Index | EMR | Body mass index recorded at the most recent encounter |
| Chronic Condition Count | EMR | Count of total chronic conditions in problem list or clinical encounter using the Charlson Comorbidity Classification |
| Chronic Kidney Disease | EMR | Problem list or clinical encounter diagnoses using standard International Classification of Diseases, Tenth Revision, Clinical Modification (ICD-10-CM), based on Charlson Comorbidity Classification |
| Diabetes | EMR | Problem list or clinical encounter diagnoses using standard International Classification of Diseases, Tenth Revision, Clinical Modification (ICD-10-CM), based on Charlson Comorbidity Classification |
| HIV/AIDS | EMR | Problem list or clinical encounter diagnoses using standard International Classification of Diseases, Tenth Revision, Clinical Modification (ICD-10-CM), based on Charlson Comorbidity Classification |
| Illicit Drug Use | EMR | Self-reported |
| Primary Care Provider Affiliation | EMR | Attributed using self-reported Primary Care Provider. Patient placed into three groups: Internal Primary Care Provider, External Primary Care Provider identified, or Unknown Primary Care Provider. |
| Polypharmacy | EMR | Number of distinct medications prescribed in the last 12 months |
| Serious Mental Illness | EMR | Major recurrent depression, schizophrenia, and bipolar disorder as defined by Charlson Comorbidity Classification |
| Substance Use Disorder | EMR | NYU (New York University) mental health psychoactive substance dependence classification |
| Dementia | EMR | Problem list or clinical encounter diagnoses using standard International Classification of Diseases, Tenth Revision, Clinical Modification (ICD-10-CM), based on Charlson Comorbidity Classification |
| Tobacco Use | EMR | Self-reported |
| Symptoms | EMR | Encounter reported fever, cough, shortness of breath, chills, myalgia |
| **Environmental** | | |
| Region | EMR | Assigned via self-reported geocoded patient address mapped to regional areas. As more patients are tested, geographical grouping can be narrowed to smaller areas. |
| Age-stratified Communal Living | EMR | Derived using age ranges and generational composition of residents clustered by geographic proximity |
| Neighborhood Financial Security | 2018 American Community Survey | Assigned via geocoded patient address using four variables: 1) Percent of population at or below 200% of the Federal Poverty Level (FPL), 2) Percent of cost burdened households where gross rent is greater than 30% of income, 3) Per capita income in the bottom quartile, and 4) Percent of households receiving SNAP benefits |
| Neighborhood Housing Security | 2018 American Community Survey | Assigned via geocoded patient address using two variables: 1) Percent of occupied housing with 1+ substandard condition such as heat, kitchen, bathroom, overcrowding, or other factors and 2) Percent housing units overcrowded |
| Neighborhood Transportation | 2018 American Community Survey | Assigned via geocoded patient address using 2 variables: 1) Percent of population using public transportation for commute to work and 2) Percent of households with no motor vehicle |
| Air Quality | Centers for Disease Control and Prevention Air Quality | Assigned via geocoded patient address using percent days exceeding standards for ozone concentration |
